# The Effect of Concentration, Temperature, and pH on the Formation of Hyaluronic Acid–Surfactant Nanohydrogels

**DOI:** 10.3390/gels9070529

**Published:** 2023-06-29

**Authors:** László Seres, Edit Csapó, Norbert Varga, Ádám Juhász

**Affiliations:** 1 Interdisciplinary Excellence Center, Department of Physical Chemistry and Materials Science, University of Szeged, H-6720 Szeged, Hungary; sereslaci8@gmail.com (L.S.); juhaszne@chem.u-szeged.hu (E.C.); 2 MTA-SZTE Lendület “Momentum” Noble Metal Nanostructures Research Group, University of Szeged, H-6720 Szeged, Hungary

**Keywords:** hyaluronic acid, surfactant, rheology, colloidal particles, hydrogel

## Abstract

The assembly of colloidal hyaluronic acid (HyA, as a polysaccharide) based hydrogel particles in an aqueous medium is characterized in the present paper, with an emphasis on the particular case of nanohydrogels formed by surfactant-neutralized polysaccharide networks. The structural changes and particle formation process of polysaccharide- and cationic-surfactant-containing systems were induced by the charge neutralization ability and the hydrophobic interactions of cetyltrimethylammonium bromide (CTAB) under different conditions. Based on the rheological, light scattering, ζ-potential, turbidity, and charge titration measurements, it can be concluded that the preparation of the HyA-CTAB particles can be greatly controlled. The results indicate that more available negative charges can be detected on the polymer chain at smaller initial amounts of HyA (c_HyA_ < 0.10 mg/mL), where a molecular solution can be formed. The change in the pH has a negligible effect on the formation process (particle aggregation appears at n_CTAB_/n_HyA,monomer_~1.0 in every case), while the temperature dependence of the critical micelle concentration (c.m.c.) of CTAB determines the complete neutralization of the forming nanohydrogels. The results of our measurements confirm that after the appearance of stable colloidal particles, a structural change and aggregation of the polymer particles take place, and finally the complete charge neutralization of the system occurs.

## 1. Introduction

Hyaluronic acid (HyA) is a naturally occurring heteropolysaccharide that can be categorized in the group of glycosaminoglycans. Because of its biocompatible and biodegradable features, it is widely used in many fields from tissue engineering to cancer therapy and from wound healing [1] to drug delivery [2]. According to its structure, it consists of two monomer units: D-glucuronic acid that contains a free carboxylic group and N-acetyl-D-glucosamine in which the acetylic group can be found in the form of an amide bond [3]. The presence of many polar and ionizable functional groups on the backbone of the macromolecule give the polymer the ability to create intermolecular hydrogen bonds with water molecules and other polymer fibers; thus, a highly hydrophilic and viscoelastic character of HyA can be observed [4]. Another consequence of the functional groups is that, according to the dissociation constant of the monomers (pK_a_, monomer ≈ 2.9 [5]), the backbone of the polymer has a net negative charge in a broad pH range.

For the preparation of the HyA-based carrier system (as hydrogels [6,7], microparticle–gel systems [8], cell microcarriers [9], nanohydrogels [10], nanoemulsions [11], core–shell carriers [12], nanoconjugates [13], etc.), the hydrophilic character of the polysaccharide needs to be modified, which can be achieved through cross-linking [14], chemical structural modifications [15], or electrostatic interactions [16]. Thanks to these methods of structural change, the encapsulation efficiency and the drug dissolution properties can be tuned, contributing to their usability in the field of biomedical research. Nowadays, one-step neutralization techniques are receiving special attention due to their feasibility, where the negatively charged HyA chain interacts with positively charged species. These neutralization methods are mainly driven by ionic interactions, and hydrophobic interactions presumably have importance as well [17], but the knowledge of these processes is still incomplete. Numerous HyA–macromolecule complexes have already been formed and studied [18], among which the most common ones are HyA–polymer (chitosan [19,20,21] and PEI [22]) and HyA–protein (zein [23], BSA [24], and HSA [16]) interactions. The obtained complexes are suitable for the delivery of small molecules, but the targeting of nucleic acids is also possible with them [25,26].

Surfactant-containing complexes are another important group of polymer-based colloidal materials [17,27,28,29,30,31]. The most common preparation of this kind of conjugate is when an ionic polymer is introduced to an oppositely charged surfactant [30]; however, a swapped experimental setup is also possible [32]. One of the greatest advantages of these systems is the solubilizing feature of the surfactant, which enables the encapsulation of hydrophobic materials, thus increasing their solubility and bioavailability. The interactions of cetyltrimethylammonium bromide (CTAB) with different polymers have already been shown, and it was proven that the systems are capable of encapsulating curcumin, an antioxidant. Prolonged effective drug release was also observed [17]; however, the sizes of the fabricated particles were mostly at the micron level. Other studies published about the interaction of CTAB with HyA reported that the surfactant was in the form of mixed [29] or individual micelles [14,28]. It was even measured that the presence of HyA can significantly decrease the cytotoxicity of CTAB [33,34,35]. Due to the negative charge of HyA and the positive charge of the surfactant, the forming interaction between the two molecules is decisively ionic. As a result of this phenomenon, the HyA loses its hydrophilicity and forms complex particles, which makes it an ideal delivery system.

**Figure 1:**
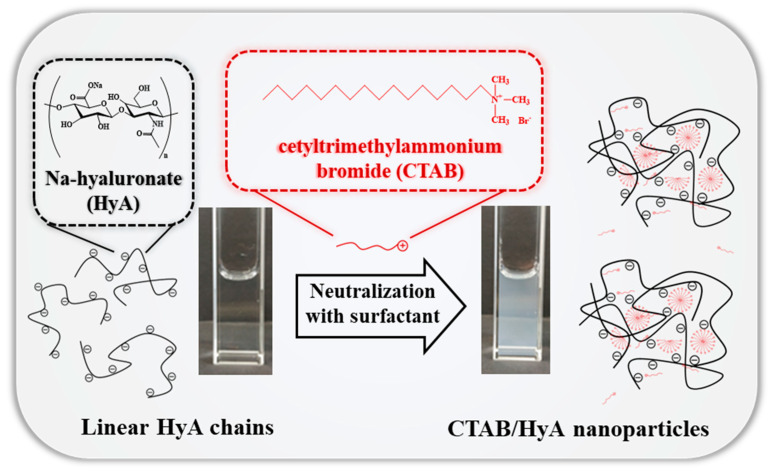
Schematic representation of the formation of the HyA-CTAB nanohydrogels.

The interaction between CTAB and HyA has already been studied, as mentioned earlier. However, the polymer concentration, pH, and temperature dependence of the particle formation have not been studied in detail yet. In addition, the analysis of the mechanism of the HyA/CTAB conjugate formation is also incomplete. Based on these observations, we determined the charge compensation processes (charge neutralization) between the negatively charged polysaccharide (HyA) and the positively charged cationic surfactant (CTAB) under different initial conditions (concentration, temperature, and pH) and the formation of the mixed-composition nanohydrogels. A schematic representation of this process can be seen in Figure 1. As a result of these measurements, we attempted to organize the main steps of the particle formation considering the rheological, optical (DLS, turbidimetry, and TEM), and charge ratio (ζ-potential and charge titration) properties of the system.

## 2. Results and Discussion

### 2.1. Size and Structural Characterization of HyA-CTAB Particles

#### 2.1.1. Effect of the Synthesis Conditions on the HyA-Based Particles

Based on the literature and our experience in the field of creating colloidal particles, the first step of our investigation was to measure the size and the size distribution the particles formed during the interaction of HyA and CTAB.

For the DLS measurements, the concentrated solution of CTAB (c_CTAB_ = 25 mM) was added dropwise to the HyA solution (c_HyA_ = 0.05–0.20 mg/mL) under 700 rpm magnetic stirring at different pH (pH = 3.6–5.5) and temperature (T = 15–35 °C) values. After one minute of stirring, the hydrodynamic diameters of the particles were registered at each titration point. Similarly, turbidimetric titrations were performed as well to follow the processes in the system. The HyA-dependence measurements were performed with the following initial parameters: c_HyA_ = 0.05, 0.10, 0.15, and 0.20 mg/mL; V_HyA_ = 10 mL; c_CTAB_ = 25 mM; V_CTAB, titration portions_ = 5–20 µL; a highly purified water medium; and T = 25 °C. pH-dependent studies were carried out as follows: c_HyA_ = 0.10 mg/mL, V_HyA_ = 10 mL, c_CTAB_ = 25 mM, V_CTAB, titration portions_ = 10 µL, pH = 3.6–5.5 acetate buffer medium, and T = 25 °C. The initial circumstances of the temperature-dependent experiments were c_HyA_ = 0.10 mg/mL, V_HyA_ = 10 mL, c_CTAB_ = 25 mM, V_CTAB, titration portions_ = 10 µL, a highly purified water medium, and T = 15–35 °C. The determination of the pH and temperature effects on the HyA/CTAB systems was performed at c_HyA_ = 0.10 mg/mL because the processes taking place in the system were more difficult to follow at higher amounts of HyA due to the formation of more particles. Furthermore, if we used a lower concentration, where the HyA-containing systems had low apparent viscosity, the comparison of the determined pH and temperature effects of the DLS results with the other measurements, such as rheology, was complicated.

**Figure 2:**
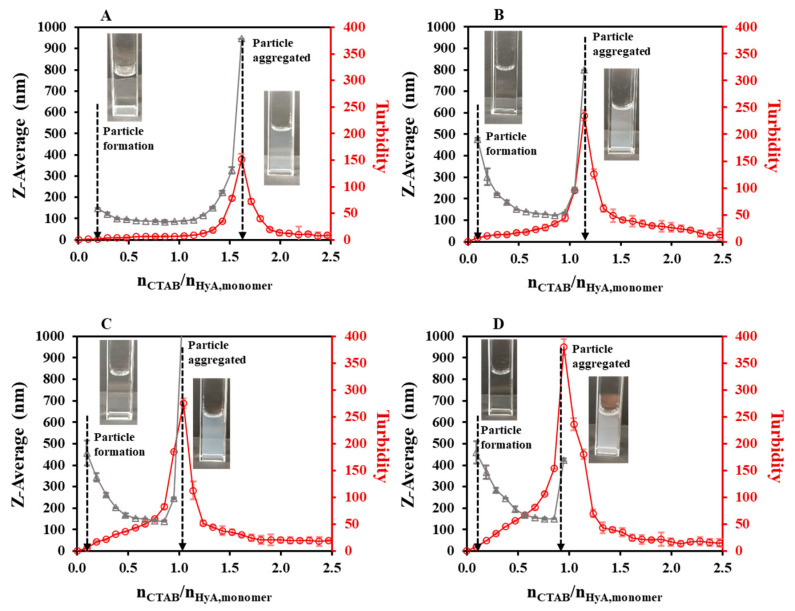
Size of the particles (△) and turbidity of the dispersion (○) during titration of HyA with CTAB (c_CTAB_ = 25 mM) at (**A**) 0.05 mg/mL, (**B**) 0.10 mg/mL, (**C**) 0.15 mg/mL, and (**D**) 0.20 mg/mL polymer concentrations (V_HyA_ = 10 mL; T = 25 °C; highly purified water medium).

As the first synthesis condition, the polymer concentration dependence of the particle size was studied (Figure 2, Figures S1A and S2). During the titrations, an initial section could be observed, where no measurable particle formation was detected. After adding more CTAB to the polymer solution, a slight decrease in the hydrodynamic size and a small increase in the turbidity values were experienced at a low concentration, while larger changes could be observed at a higher HyA concentration. Then, a nearly constant region was observed, and after the addition of a certain amount of surfactant, the aggregation of the particles occurred. When examining the particle size in the constant regions of the curves, the increment in the diameter could be observed as the concentration of the HyA increased (from 85.7 nm (c_HyA_ = 0.05 mg/mL) to 150 nm (c_HyA_ = 0.20 mg/mL) at n_CTAB_/n_HyA,monomer_ = 0.86). The most important feature of the system, besides the particle size, is the molar ratio at the aggregation point. While increasing the concentration of the polymer, a decrease in the CTAB/HyA monomer molar ratio at the aggregation point was measured.

At low concentrations of the polymer, where a molecular solution could be formed [14], more negative charges were available to neutralize with CTAB; thus, smaller particles were formed and more CTAB was needed to reach the aggregation point (at 0.05 mg/mL, the aggregation point was at n_CTAB_/n_HyA,monomer_ = 1.62). In contrast to the higher concentrations, where a coherent structure could be formed, the precipitation of the particles happened sooner (at 0.20 mg/mL, the aggregation point was at n_CTAB_/n_HyA,monomer_ = 0.95) since the formation of bigger particles happened and more functional groups were closed into the internal part of the particle, thus shadowing them from the surfactant. These results were also confirmed by turbidimetric measurements (Figure 2). The maximum points of the turbidity curves exactly matched the highest values of the DLS measurements, which is a proof that aggregation really occurred at those points. If more CTAB was added to the medium, a decrease in the turbidity was experienced, which was due to the sedimentation of bigger aggregates and, thus, the clearance of the dispersion.

**Figure 3:**
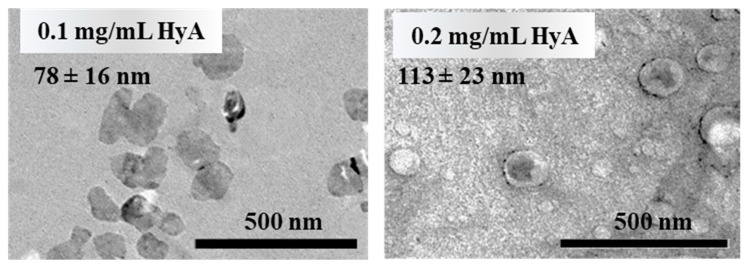
Representative TEM images of the prepared nanoparticles at different HyA concentrations (n_CTAB_/n_HyA,monomer_ = 0.8, highly purified water medium).

For further characterization of the system, TEM images were recorded of the particles at an n_CTAB_/n_HyA,monomer_ = 0.8 molar ratio (Figure 3). The images clearly show that in these conditions, amorphous particles can be synthetized, and the results are in good agreement with the DLS measurements.

**Figure 4:**
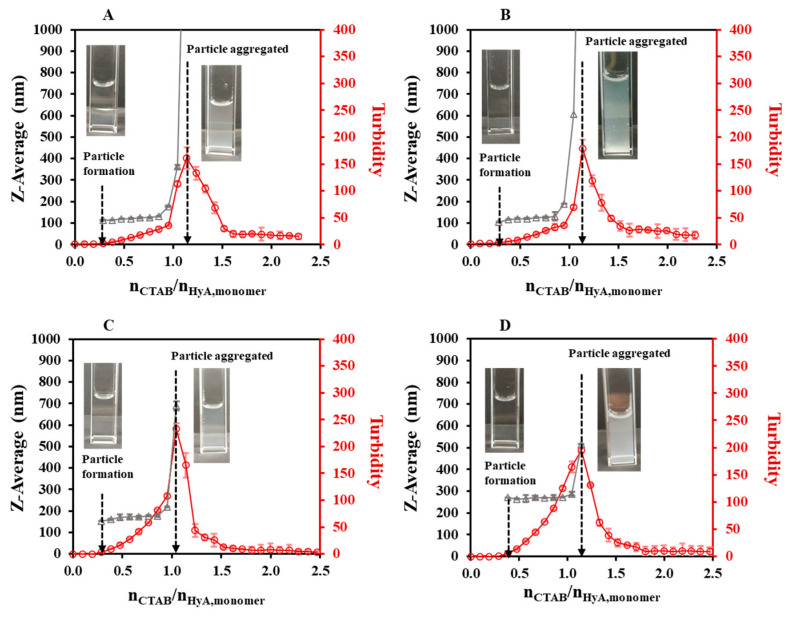
Size of the particles (△) and turbidity of the dispersion (○) during titration of HyA with CTAB (c_CTAB_ = 25 mM) in (**A**) pH = 3.6, (**B**) pH = 4.0, (**C**) pH = 4.5, and (**D**) pH = 5.5 acetate buffer media (c_HyA_ = 0.10 mg/mL, V_HyA_ = 10 mL, T = 25 °C).

As a continuation of our experiments, pH-dependent light scattering studies were carried out in the pH = 3.6–5.5 range (Figure 4, Figures S1B and S3). Figure 4 shows that the HyA-CTAB particles appeared later in the acetate buffer medium than in the previously investigated highly purified water medium (Figure 2). If we take into consideration the particle size values of the initial quasi-constant sections, at pH = 3.6 and 4.0, particles of approx. 125 nm were synthesized; however, at pH = 4.5 the hydrodynamic diameter of the particles increased to ~173 nm, and a further increase was observed at pH = 5.5 to ~265 nm. Such a systematic change in the molar ratio values at the aggregation points (n_CTAB_/n_HyA,monomer_ = 1.05–1.14) could not be observed, which means that in the case of aggregation, pH does not play a key role. As expected, the turbidity of the dispersions increased with the addition of CTAB, and it can be clearly said that the particle formation started where the slope of the turbidity curves was about to increase, which matched the first observable particles in the DLS measurements.

**Figure 5:**
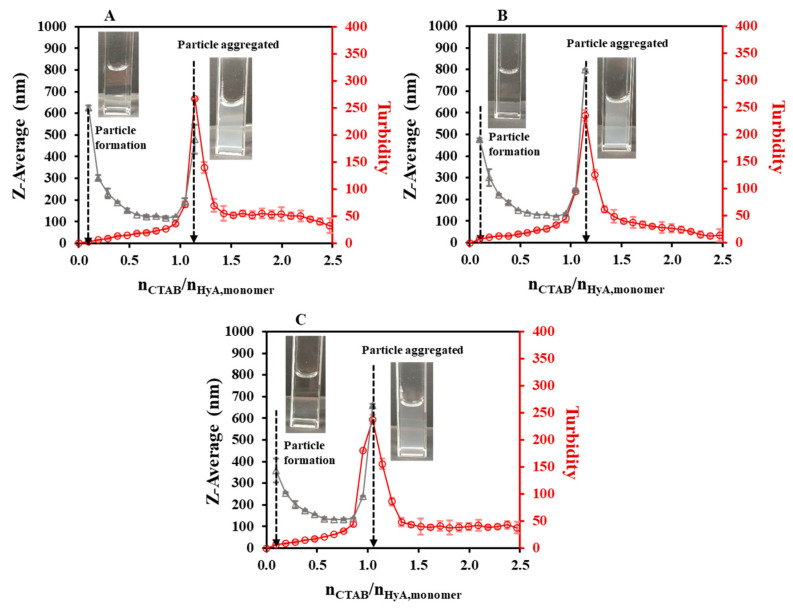
Size of the particles (△) and turbidity of the dispersion (○) during titration of HyA with CTAB (25 mM) at (**A**) 15 °C, (**B**) 25 °C, and (**C**) 35 °C temperatures (c_HyA_ = 0.10 mg/mL, V_HyA_ = 10 mL, highly purified water medium).

The final step of light scattering characterization was the temperature-dependent studies. As Figure 5 shows, quite similar particle size and turbidimetric profiles were measured at a 0.1 mg/mL HyA concentration (Figures S1C and S4). A great decrease in the particle sizes could be observed in the initial section of the curves (like in the concentration-dependent studies). Before the start of the aggregation, the particle size values increased from ~118 nm (T = 15 °C) to ~133 nm (T = 35 °C). The molar ratio at the aggregation point did not vary significantly (n_CTAB_/n_HyA,monomer_ = 1.05–1.14); however, the starting aggregation point was slightly decreased (n_CTAB_/n_HyA,monomer_ = 1.02 (T = 15 °C), 1.02 (T = 25 °C), and 0.93 (T = 35 °C) with DLS; n_CTAB_/n_HyA,monomer_ = 1.02 (T = 15 °C), 0.95 (T = 25 °C), and 0.83 (T = 35 °C) with turbidity). Therefore, it can be concluded that the temperature influenced the formation of the HyA-CTAB systems in the observed temperature range.

#### 2.1.2. Zeta-Potential of HyA-CTAB Particles

**Figure 6:**
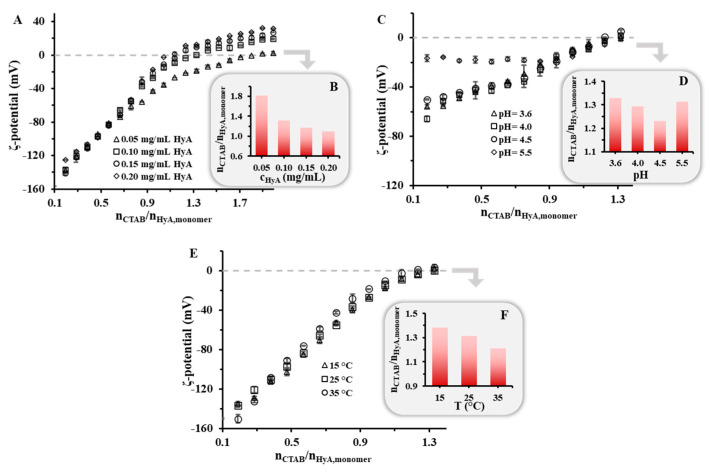
Zeta-potential values of the HyA/CTAB system during titration of HyA with CTAB (25 mM) at different (**A**) polymer concentrations (c_HyA_ = 0.05–0.20 mg/mL; V_HyA_ = 10 mL; highly purified water medium), (**C**) pH values (c_HyA_ = 0.10 mg/mL; V_HyA_ = 10 mL; pH = 3.6–5.5 acetate buffer media), and (**E**) temperatures (c_HyA_ = 0.10 mg/mL; V_HyA_ = 10 mL; T = 15–35 °C; highly purified water medium). (**B**,**D**,**F**) The diagrams on the bottom right indicate the n_CTAB_/n_HyA,monomer_ values at 0 mV ζ-potential.

Besides the particle size, the charge of the macromolecule–surfactant nanohydrogels is a determining factor in the preparation of kinetically stable colloidal particles. To map the charge ratios of the system, the formerly presented titrations were carried out while measuring the ζ-potential of the particles in different experimental conditions, where the same synthesis parameters were used as for the DLS and turbidity measurements (Figure 6). In all the experiments, during the first steps very low ζ-potential values were measured, which was due to the excess of the negatively charged polymer. Upon the addition of the positively charged CTAB, the ζ-potential values started to increase, and at a certain amount, the complete neutralization of the system was achieved (where the ζ-potential = 0 mV), after which the electrokinetic potential was only determined by the excess surfactant. First, the HyA concentration dependence was studied in a highly purified water medium. When examining the curves, it was clearly visible that the neutralization point shifted to lower values with increasing HyA amounts in the solution, which confirmed the DLS and turbidity measurements (Figure 1), according to which more negative functional groups were available for the surfactant at a lower HyA concentration. In the case of pH (Figure 6C,D), an exact tendency could not be observed in the shift of the surfactant/polymer molar ratios at the neutralization point. The main reason for the different values and trends was the different ionic strength of the buffer media since with increasing pH the ionic strength of the buffer solution increased as well. When studying the temperature dependence of the system, a slight decrease in the neutralization points could be observed (n_CTAB_/n_HyA,monomer_ = 1.38 (T = 15 °C), 1.31 (T = 25 °C), and 1.21 (T = 35 °C)), similar to the start of the aggregation points determined by DLS and turbidity (Figure 5). This can presumably be explained by the increase in the CTAB c.m.c. (Figure S5). The formation of partial micelles is less favorable at higher temperatures; thus, one negative charge can more likely be neutralized with an individual CTAB molecule than with a micelle aggregate, resulting in a decrease in the neutralization point.

### 2.2. Study of the HyA-CTAB Interaction

#### 2.2.1. Rheology

**Figure 7:**
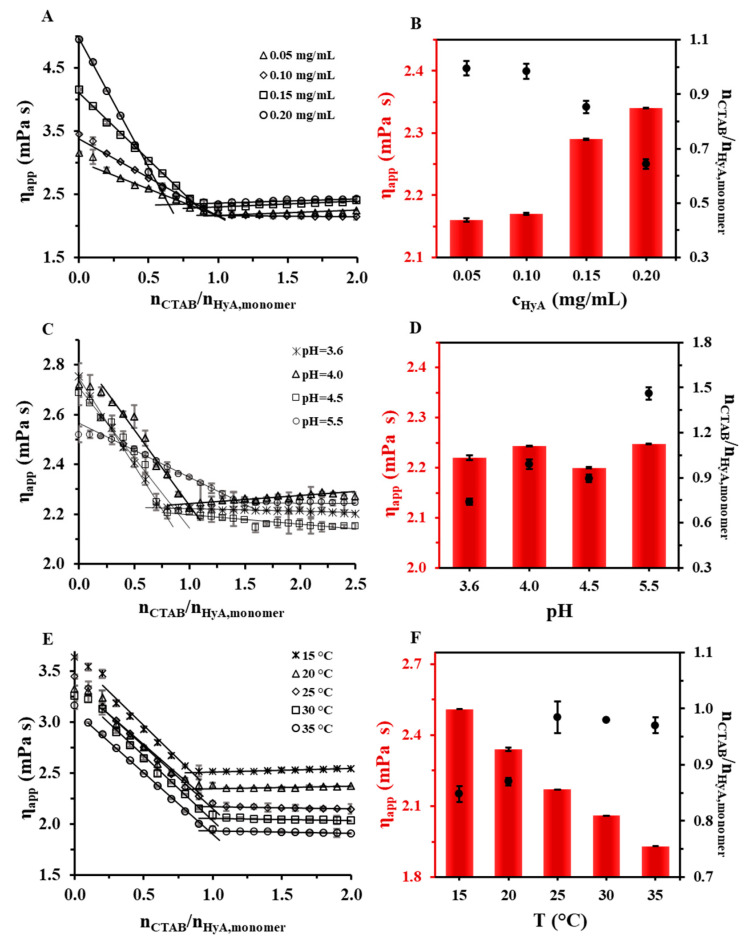
Rheological measurements of HyA titrated with 25 mM CTAB at different (**A**) polymer concentrations (c_HyA_ = 0.05–0.20 mg/mL; V_HyA_ = 19 mL; highly purified water medium), (**C**) pH values (c_HyA_ = 0.10 mg/mL; V_HyA_ = 19 mL; pH = 3.6–5.5 acetate buffer media), and (**E**) temperatures (c_HyA_ = 0.10 mg/mL; V_HyA_ = 19 mL; T = 15–35 °C; highly purified water medium). Diagrams (**B**,**D**,**F**) show the apparent viscosity and n_CTAB_/n_HyA,monomer_ molar ratios at the breaking points of the curves.

Since HyA is a hydrophilic polymer that swells very well in aquatic media, creating a gel-like structure, we investigated its behavior with rheology examinations. The experimental setup was similar to the light scattering and ζ-potential measurements: titration was executed, and the apparent viscosity value of the system was registered. The titrations were performed at different HyA concentrations, pH values, and temperatures, as can be seen in Figure 7. As a general observation of the measurements, it can be stated that the addition of a cationic surfactant to the HyA solution decreased the viscosity of the solution, which can be explained by the collapse of the coherent structure of HyA. After the addition of a certain amount of CTAB, no more decrease in viscosity could be experienced, and a breaking point could be noticed in the curves since the cross-linked structure of the polymer had entirely collapsed. This characteristic point provided us quantitative information. After that, constant or slightly increasing viscosity values were measured.

When examining the polymer concentration dependence of the system (Figure 7A,B), it is clear that increasing the concentration of HyA lead to a decrease in the surfactant/polymer monomer molar ratios and an increase in the apparent viscosity at the intersections of the characteristic linear sections (from 0.995 (0.05 mg/mL) to 0.644 (0.20 mg/mL), Figure 7B). This molar ratio change showed similarities with the light scattering techniques and the ζ-potential determination measurements, where the same decreasing trend was observed at the aggregation points as well as at the neutralization points (Figure 2 and Figure 6A). It turned out that the breaking point happened at similar molar ratios as the start of the aggregation of HyA-CTAB particles (n_CTAB_/n_HyA,monomer_ = 1.02 (T = 15 °C), 1.02 (T = 25 °C), and 0.93 (T = 35 °C) with DLS; n_CTAB_/n_HyA,monomer_ = 1.02 (T = 15 °C), 0.95 (T = 25 °C), and 0.83 (T = 35 °C) with turbidity). When examining the pH of the medium (Figure 7C,D), first the protonation possibilities of the two materials needed to be considered. In the investigated pH range, HyA is practically entirely deprotonated and thus has a negative charge (pK_a,HyA_ ≈ 2.9 [5,36]), while CTAB has a positive charge in the full range. These facts predicted no pH dependence of the interaction of the molecules; nonetheless, a significant increase in the surfactant/polymer monomer molar ratios at the breaking points could be seen with an increasing pH. The main reason for this change must be the ionic strength dependence of the system because significantly different amounts of buffer salts were dissolved in the media (I = 0.18 mM (pH = 3.6), 0.45 mM (pH = 4.0), 5.69 mM (pH = 4.5), and 56.9 mM (pH = 5.5)). With this explanation, the increment in the ionic strength is affected by the surfactant/polymer monomer molar ratio values at the breaking points. In the temperature-dependent studies, a clearly observable decreasing trend in the apparent viscosity values at the breaking points could be seen, as expected, while the increase in the surfactant/polymer monomer molar ratio values at the breaking points could be observed from n_CTAB_/n_HyA,monomer_~0.85 (T = 15 °C) to n_CTAB_/n_HyA,monomer_~0.97 (T = 35 °C). These results confirmed that the temperature also influenced the structural changes of the HyA, in addition to the aggregation processes of the HyA-CTAB particles and the charge neutralization of the HyA.

#### 2.2.2. Charge Titration

Based on the results of the ζ-potential measurements and the assumption that the interaction between the two molecules is mainly driven by Coulombic forces, we wanted to further strengthen the electrostatic features of the system, so charge titration studies were carried out as well. Charge titration measurements were performed in similar conditions as the previous measurements.

**Figure 8:**
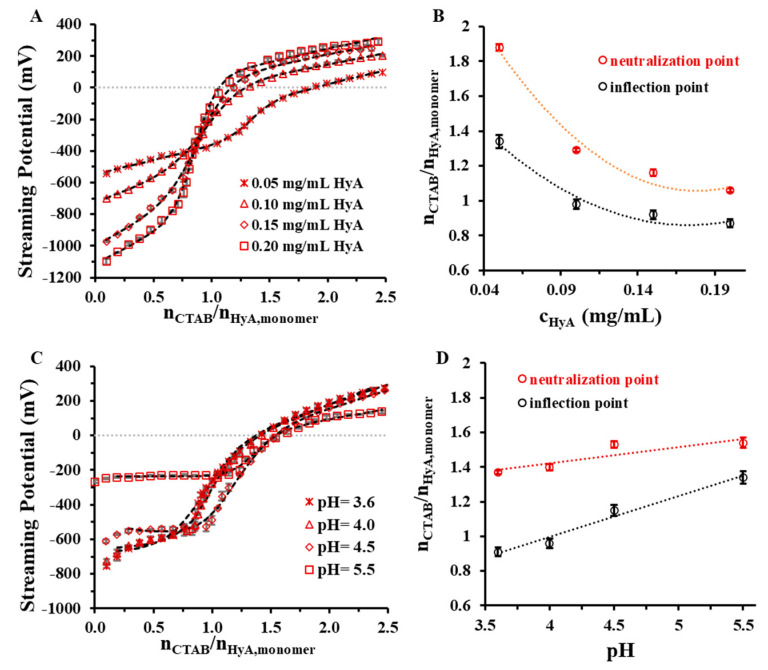
Charge titration measurements of HyA titrated with CTAB at different (**A**) polymer concentrations (c_HyA_ = 0.05–0.20 mg/mL; V_HyA_ = 10 mL; highly purified water medium) and (**C**) pH values (c_HyA_ = 0.10 mg/mL; V_HyA_ = 10 mL; pH = 3.6–5.5 acetate buffer media). Diagrams (**B**,**D**) show the n_CTAB_/n_HyA,monomer_ molar ratios at the PZC points and inflection points of the curves.

Figure 8 shows the charge titration curves at different HyA concentrations and pH values.

The polymer concentration dependence shows that the increasing amount of HyA in the aqueous medium decreased the initial streaming potential of the system, which can be explained by the higher number of negative charges in the medium. All the titration curves follow a sigmoidal profile and intersect each other near the inflection point, except the 0.05 mg/mL macromolecule solution. Consistent with the ζ-potential, DLS, turbidity, and rheology measurements, the 0 mV streaming potential was reached at higher surfactant/macromolecule monomer molar ratios (Figure 8B and Table 1). When examining the pH dependence of the system, a slight increase in the neutralization and inflection points was observable with an increasing pH (Figure 8D), which might have been the consequence of the increment in the ionic strength, as was stated earlier in the rheology measurements. The opposite explanation is valid for the starting points of the curves: as the pH (and ionic strength) increased, the streaming potential values decreased due to the shadowing of the ions.

In order to compare the CTAB/HyA monomer molar ratios determined during certain measurements and to characterize the role of charge compensation in the synthesis process, the results are summarized in Table 1. It can be seen that the neutralization of the HyA (streaming potential was 0 mV) occurred later than the aggregation of the HyA-CTAB particles, independent of the synthesis condition; thus, the destabilization of the homogenous HyA-CTAB systems took place before the charge compensation process. It also can be stated that the structural change in the HyA (from the rheology measurements) appeared to be mostly parallel to the start of the aggregation (DLS and turbidity). With this knowledge, it can be concluded that the steps following the formation of the particles are the structural change, the aggregation process, and finally the neutralization of the system.

## 3. Conclusions

Different characterization techniques were used to investigate the formation of colloidal particles from the assembly of HyA and CTAB. Via the specifications of these measuring methods and the applied temperature-, pH-, and polymer-concentration-dependent titrations, the characteristic structural changes of the complex colloid system and the process of particle formation were identified. HyA-concentration-dependent studies revealed that increasing the amount of polymer in the system results in the formation of larger particles, although the charge neutralization points shift to lower surfactant/polymer monomer molar ratios. This may be due to the increasingly molecular-solution-like behavior of the system, which results in fewer shadowed negative charges during particle formation. pH-dependent experiments showed that the acidity is not a significant determining factor, although the ionic strength of the medium must be considered for the preparation of the particles since the hydrodynamic diameter of the particles can vary significantly with the amount of salt dissolved in the medium. The effect of temperature on the particles can also be stated, which means that the processes in the HyA-CTAB systems can be tuned with this parameter. As the final step of our investigation, we suggest an apparent mechanism of the interaction between CTAB and HyA. During the addition of the surfactant, the formation of colloidal nanohydrogels can be determined first. After that, HyA loses its structure and the aggregation of HyA-CTAB systems starts. The complete charge neutralization of the system only takes place after these processes.

## 4. Materials and Methods

### 4.1. Materials

Hyaluronic acid sodium salt (HyA, 1.5–1.8⋅10^6^ Da) and cetyltrimethylammonium bromide (CTAB, CH_3_(CH_2_)_15_N(Br)(CH_3_)_3_; 95%) were obtained from Sigma-Aldrich, Hungary. Sodium acetate 3-hydrate (CH_3_COONa⋅3H_2_O; ≥99%) and acetic acid (CH_3_COOH, ≥99%) were purchased from Molar Chemicals, Hungary. Highly purified water was obtained by deionization and filtration with a Millipore purification apparatus (18.2 MΩ·cm at 25 °C). All solvents and reagents used for preparation were of analytical grade, and no further purifications were made.

### 4.2. Methods

#### 4.2.1. Preparation of the CTAB-Modified HyA (HyA/CTAB) Particles

The HyA/CTAB systems were prepared using the charge neutralization method, where the formation of the NPs was studied in a 0.09–2.85 CTAB/HyA monomer molar ratio. Depending on the synthesis conditions, the HyA (c_HyA_ = 1 mg/mL) and CTAB (c_CTAB_ = 25 mM) were dissolved in an acetate buffer (pH = 3.6–5.5) or MilliQ water. Before starting the NP synthesis, the HyA stock solution was stirred at 300 rpm for 30 min and stored for 24 h at 10 °C. The HyA solution was diluted to 0.05–0.20 mg/mL (10 mL), and the CTAB stock solution was added dropwise in 5–20 µL portions to the HyA solution under 700 rpm magnetic stirring. Samples were mixed for at least 5 min before further use. The HyA and CTAB concentrations, media, and temperature for each sample series are shown in Table 2.

#### 4.2.2. Rheological Measurements

Rheological studies were carried out with the help of an Anton Paar Physica MCR 301 Rheometer (Anton Paar, GmbH, Ostfildern, Germany). The measuring system was equipped with a cylinder with a concentric geometry (CC27-SN12793) and a Peltier heating system, as well as with a water bath, to enable temperature-dependent measurements. The apparent viscosity values of the CTAB/HyA systems were determined at a 300 1/s shear rate, and the values were studied under different conditions: c_HyA_ = 0.05–0.20 mg/mL, T = 10–40 °C, and a pH = 3.6–5.5 acetate buffer and a highly purified water medium. A CTAB (25 mM) solution was added dropwise in a 19 mL HyA solution with a 10–40 µL/min dosing speed. The effect of the dilution of HyA was also measured (Figures S6–S8).

#### 4.2.3. Particle Charge Detector (PCD)

The charge titration curves of the CTAB/HyA systems were determined using a Mütek Particle Charge Detector PCD-04 model (BTG Instruments GmbH, Weßling, Germany). A volume of 10 mL of a diluted HyA solution was titrated with a 25 mM CTAB solution at 25 °C, and the streaming potential value of the system was recorded. During the characterization, 0.05–0.20 mg/mL HyA concentrations and acetate buffers with different pH values (pH = 3.6–5.5) were used. The measured curves were fitted by a modified 6-parameter Boltzmann equation using a non-linear regression [19]:

(1) $$U_{str}=\frac{a_{1}x+a_{2}}{1+\exp[\left(x-a_{4}\right)/a_{3}}+a_{5}x+a_{6}$$

where *x* represents the molar ratio of CTAB to the HyA monomer (n_CTAB_/n_HyA,monomer_), *U_str_* is the measured streaming potential of the system in one titration step, *a*_1_ and *a*_2_ are the asymptotic values of the initial and final parts of the curves, *a*_3_ measures the width of the transition zone, *a*_4_ gives the inflection point, and *a*_5_ and *a*_6_ are the slope and the intercept of the final section of the curve. With this form of the Boltzmann equation, it becomes possible to calculate the n_CTAB_/n_HyA,monomer_ molar ratio at *the U_str_ =* 0 mV point.

#### 4.2.4. Characterization of the HyA/CTAB NPs

Dynamic light scattering (DLS) and ζ-potential measurements were carried out with a HORIBA SZ-100 NanoParticle Analyzer (HORIBA Jobin Yvon, Longjumeau, France) equipped with a semiconductor laser (λ = 532 nm, 10 mW) as a light source and a photomultiplier detector for the quantification of scattered intensity at a 90° scattering angle. As a result, the hydrodynamic diameter, size distribution, and zeta-potential of the particles could be measured. The transmission electron microscopic (TEM) images were recorded by Jeol JEM-1400plus equipment (JEOL Ltd., Tokyo, Japan) at 120 keV acceleration. A turbidimetric analysis was performed using Precision Bench Turbidity Meter LP2000 (Hanna Ins., Budapest, Hungary) equipment. Conductometric titrations were performed to determine the critical micelle concentrations of CTAB in different media (Figure S9). To register the conductance of the solutions, Metrohm 912 conductometer (Metrohm Hungary Kft., Budapest, Hungary) equipment was used, and the buffer was titrated with a 25 mM CTAB solution. For the determination of the c.m.c. value of the surfactant, a linear regression was used on the two characteristic parts of the curves.

## Figures and Tables

**Table 1:** Summary of the characteristic points of different measurement techniques for the HyA/CTAB system.

			Rheology	DLS		Turbidity		ζ-Potential	Charge Titration
c_HyA_ (mg/mL)	T (°C)	pH	HyA Structure Change (n_CTAB_/n_HyA,monomer_)	Starting Particle Aggregation (n_CTAB_/n_HyA,monomer_)	Particle Aggregation Max. (n_CTAB_/n_HyA,monomer_)	Starting Particle Aggregation (n_CTAB_/n_HyA,monomer_)	Particle Aggregation Max. (n_CTAB_/n_HyA,monomer_)	Neutralization (n_CTAB_/n_HyA,monomer_)	Neutralization (n_CTAB_/n_HyA,monomer_)
0.05	25	-	1.00 ± 0.03	1.26	1.62	1.39	1.62	1.81	1.88 ± 0.02
0.10	25	-	0.98 ± 0.03	1.02	1.14	0.95	1.14	1.31	1.29 ± 0.01
0.15	25	-	0.85 ± 0.02	0.94	1.05	0.83	1.05	1.17	1.16 ± 0.02
0.20	25	-	0.64 ± 0.02	0.85	0.95	0.77	0.95	1.10	1.06 ± 0.01
0.10	25	3.6	0.74 ± 0.02	0.93	1.14	0.94	1.14	1.33	1.37 ± 0.01
0.10	25	4.0	0.99 ± 0.03	0.94	1.14	1.02	1.14	1.29	1.40 ± 0.02
0.10	25	4.5	0.90 ± 0.03	0.94	1.05	0.92	1.05	1.23	1.53 ± 0.02
0.10	25	5.5	1.46 ± 0.04	1.04	1.14	0.82	1.14	1.31	1.54 ± 0.03
0.10	15	-	0.85 ± 0.01	1.02	1.14	1.02	1.14	1.38	-
0.10	20	-	0.87 ± 0.01	-	-	-	-	-	-
0.10	25	-	0.98 ± 0.03	1.02	1.14	0.95	1.14	1.31	-
0.10	30	-	0.98 ± 0.02	-	-	-	-	-	-
0.10	35	-	0.97 ± 0.02	0.93	1.05	0.83	1.05	1.21	-

**Table 2:** The compositions of samples used in the experiments.

	T (°C)	Medium	c_HyA_ (mg/mL)	c_CTAB_ (mM)
HyA concentration dependence	25	water/pH = 4.5 (acetate buffer)	0.05	0.013–0.37
	25	water/pH = 4.5 (acetate buffer)	0.10	0.025–0.73
	25	water/pH = 4.5 (acetate buffer)	0.15	0.037–1.08
	25	water/pH = 4.5 (acetate buffer)	0.20	0.050–1.42
Temperature dependence	15	water	0.10	0.025–0.73
	20	water	0.10	0.025–0.73
	25	water	0.10	0.025–0.73
	30	water	0.10	0.025–0.73
	35	water	0.10	0.025–0.73
Medium dependence	25	pH = 3.6 (acetate buffer)	0.10	0.025–0.73
	25	pH = 4.0 (acetate buffer)	0.10	0.025–0.73
	25	pH = 4.5 (acetate buffer)	0.10	0.025–0.73
	25	pH = 5.5 (acetate buffer)	0.10	0.025–0.73

## Data Availability

The data are contained within the article and the Supplementary Material.

## Supplementary Materials

The following are available online at https://www.mdpi.com/article/10.3390/gels9070529/s1, Figure S1: The differential distribution curves of HyA/CTAB particles (n_CTAB_/n_HyA_ = 0.76) at (A) different HyA concentrations (c_HyA_ = 0.05–0.20 mg/mL, T = 25 °C, highly purified water medium), (B) different pH values (c_HyA_ = 0.10 mg/mL, T = 25 °C, pH = 3.6–5.5 acetate buffer medium), and (C) different temperatures (c_HyA_ = 0.10 mg/mL, T = 15–25 °C, highly purified water medium); Figure S2: Polydispersity of the dispersion during titration of HyA with CTAB (c_CTAB_ = 25 mM) at (A) 0.05 mg/mL, (B) 0.10 mg/mL, (C) 0.15 mg/mL, and (D) 0.20 mg/mL polymer concentrations (V_HyA_ = 10 mL; T = 25 °C; highly purified water medium); Figure S3: Polydispersity of the dispersion during titration of HyA with CTAB (c_CTAB_ = 25 mM) in (A) pH = 3.6, (B) pH = 4.0, (C) pH = 4.5, and (D) pH = 5.5 acetate buffer media (c_HyA_ = 0.10 mg/mL, V_HyA_ = 10 mL, T = 25 °C); Figure S4: Polydispersity of the dispersion during titration of HyA with CTAB (c_CTAB_ = 25 mM) at (A) 15 °C, (B) 25 °C, and (C) 35 °C temperatures (c_HyA_ = 0.10 mg/mL, V_HyA_ = 10 mL, highly purified water medium); Figure S5: c.m.c. of the CTAB (c_CTAB_ = 25 mM) at different temperatures (c_HyA_ = 0.10 mg/mL, T = 15–35 °C, highly purified water medium); Figure S6: The apparent viscosity curves of the HyA at (A) 0.05 mg/mL, (B) 0.10 mg/mL, (C) 0.15 mg/mL, and (D) 0.20 mg/mL (filled markers: dilution; empty markers: uncorrected and CTAB-titrated HyA solution) (V_HyA_ = 19 mL, c_CTAB_ = 25 mM, highly purified water medium); Figure S7: The apparent viscosity curves of the HyA at (A) pH = 3.6, (B) pH = 4.0, (C) pH = 4.5, and (D) pH = 5.5 acetate buffer (filled markers: dilution; empty markers: uncorrected and CTAB-titrated HyA solution) (c_HyA_ = 0.10 mg/mL; V_HyA_ = 19 mL; c_CTAB_ = 25 mM); Figure S8: The apparent viscosity curves of the HyA at (A) 15 °C, (B) 20 °C, (C) 25 °C, (D) 30 °C, and (E) 35 °C (filled markers: dilution; empty markers: uncorrected and CTAB-titrated HyA solution) (c_HyA_ = 0.10 mg/mL, V_HyA_ = 19 mL, c_CTAB_ = 25 mM, highly purified water medium); Figure S9: The conductometric titration of highly purified water with CTAB (25 mM) at (A) 15 °C, (B) 20 °C, (C) 25 °C, (D) 30 °C, and (E) 35 °C temperatures.
