# Supplementary material for: The Effect of Concentration, Temperature, and pH on the Formation of Hyaluronic Acid–Surfactant Nanohydrogels
Source: Gels. 2023 Jun 29;9(7):529. doi: 10.3390/gels9070529 (PMC10379036; doi:10.3390/gels9070529)
Supplement: Supplementary file 1 [file gels-09-00529-s001.zip › gels-2449457-supplementary.pdf]

gels

Supplementary Materials:

The Effect of Concentration, Temperature, and pH on the Formation of Hyaluronic Acid–Surfactant Nanohydrogels

László Seres [1,2], Edit Csapó [1,2], Norbert Varga [2,*] and Ádám Juhász [1,*]

1 Interdisciplinary Excellence Center, Department of Physical Chemistry and Materials Science, University of Szeged, H-6720 Szeged, Hungary; sereslaci8@gmail.com (L.S.); juhaszne@chem.u-szeged.hu (E.C.)

2 MTA-SZTE Lendület "Momentum" Noble Metal Nanostructures Research Group, University of Szeged, H-6720 Szeged, Hungary

* Correspondence: vargano@chem.u-szeged.hu (N.V.); juhaszad@chem.u-szeged.hu (Á.J.); Tel.: +36-(62)-544-211 (N.V. & Á.J.)

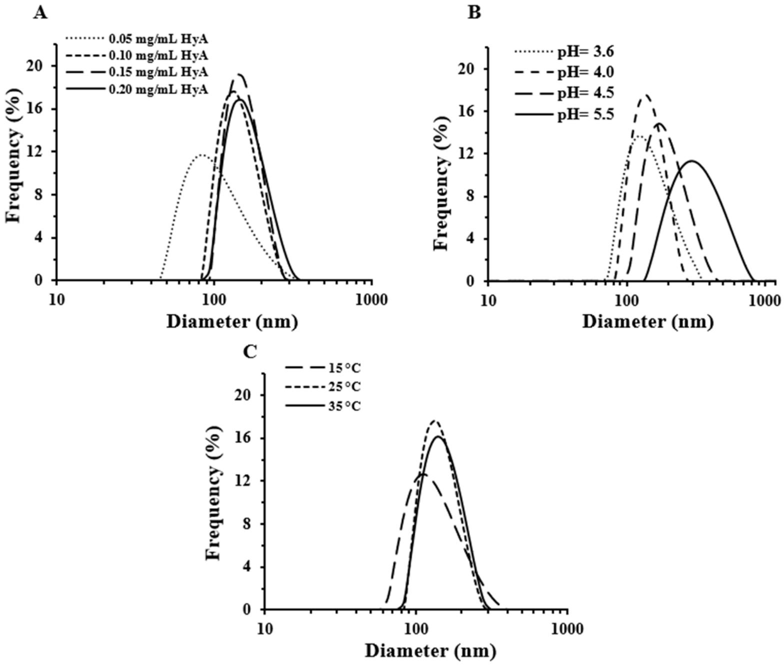

Figure S1. The differential distribution curves of HyA/CTAB particles (n_{CTAB}/n_{HyA}=0.76) at (**A**) different HyA concentrations (c_{HyA} = 0.05 – 0.20 mg/mL, T = 25 °C, high purified water medium) (**B**) different pH values (c_{HyA} = 0.1 mg/mL, T = 25 °C, pH = 3.6 - 5.5 acetate buffer medium) and (**C**) different temperatures (c_{HyA} = 0.10 mg/mL, T = 15-25 °C, high purified water medium).

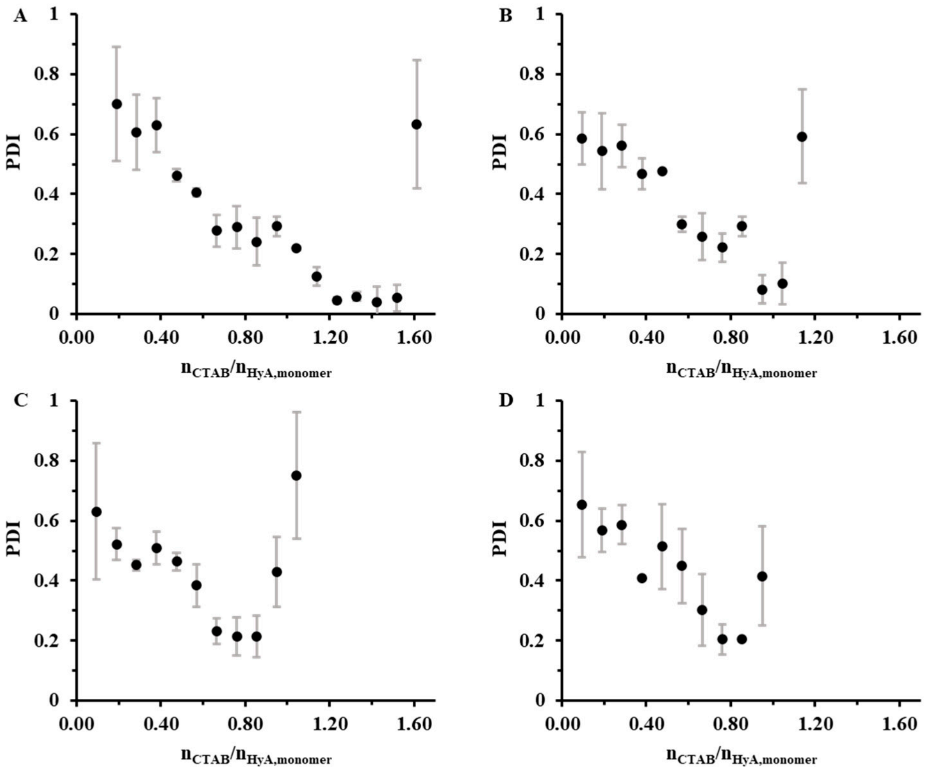

Figure S2. Polydispersity of the dispersion during titration of HyA with CTAB (c_{CTAB} = 25 mM) at (**A**) 0.05 mg/mL, (**B**) 0.10 mg/mL, (**C**) 0.15 mg/mL and (**D**) 0.20 mg/mL polymer concentrations (V_{HyA} = 10 mL; T = 25 °C; high purified water medium).

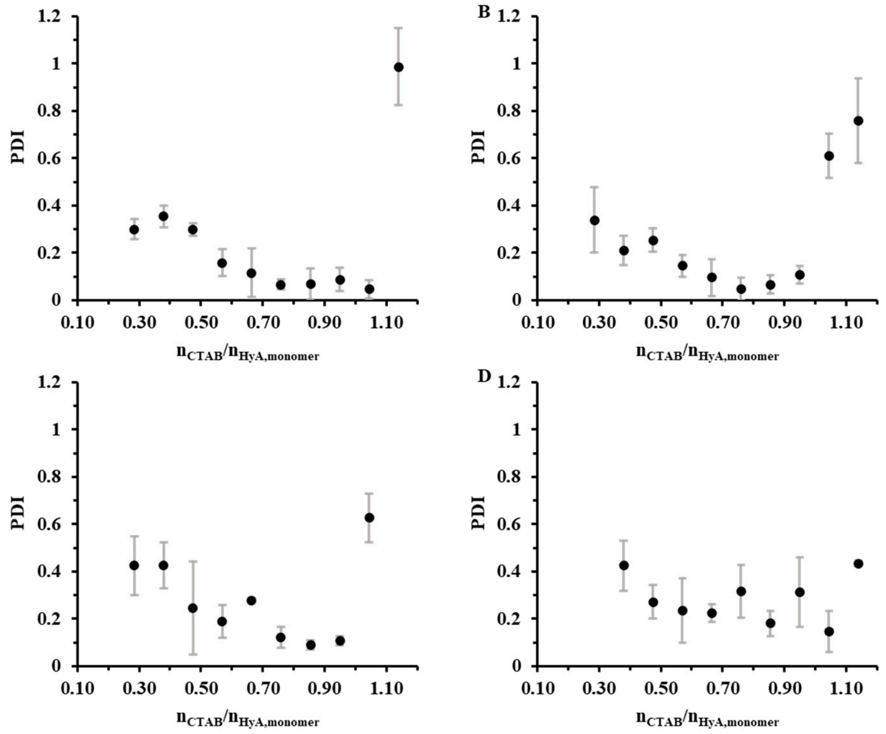

Figure S3. Polydispersity of the dispersion during titration of HyA with CTAB (c_{CTAB} = 25 mM) in (**A**) pH=3.6, (**B**) pH=4.0, (**C**) pH=4.5 and (**D**) pH=5.5 acetate buffer media (c_{HyA} = 0.10 mg/mL, V_{HyA} = 10 mL; T = 25°C).

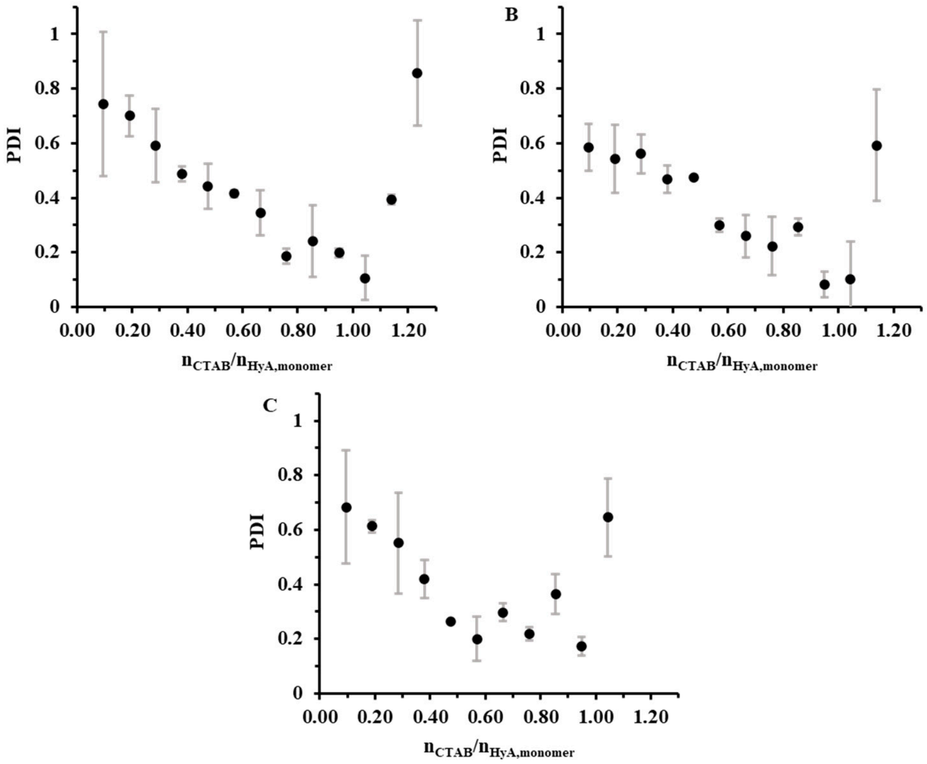

Figure S4. Polydispersity of the dispersion during titration of HyA with CTAB (c_{CTAB} = 25 mM) at (**A**) 15°C, (**B**) 25°C and (**C**) 35°C temperatures (c_{HyA} = 0.10 mg/mL, V_{HyA} = 10 mL; high purified water medium).

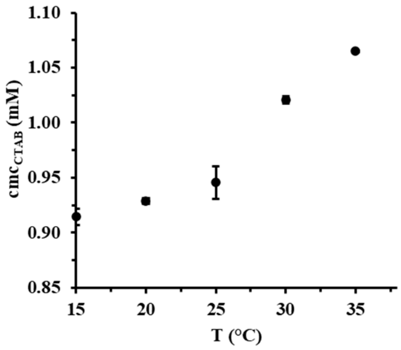

Figure S5. c.m.c. of the CTAB (c_{CTAB} = 25 mM) at different temperatures (c_{HyA} = 0.10 mg/mL, T = 15 – 35 °C, high purified water medium).

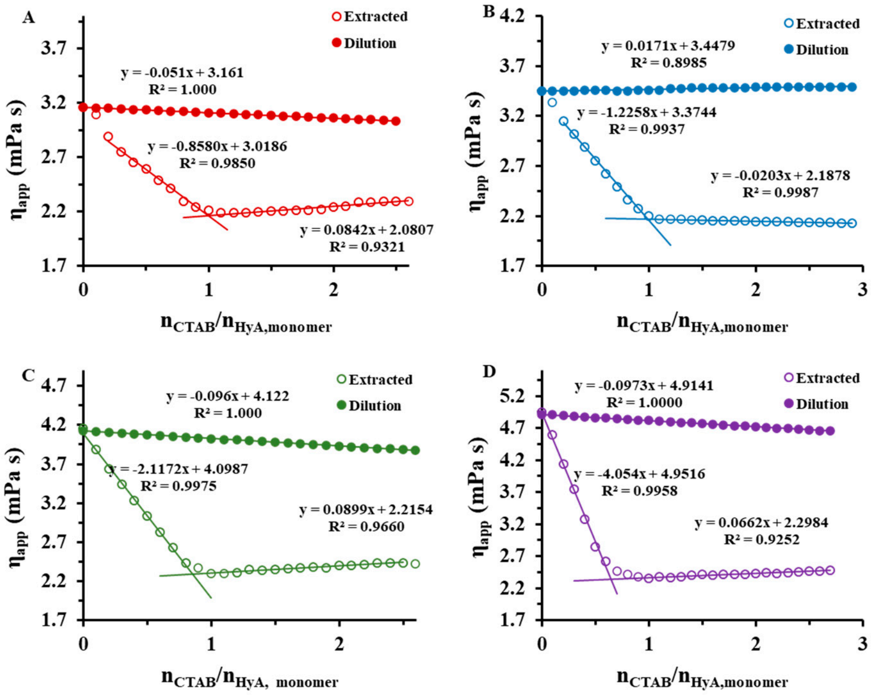

Figure S6. The apparent viscosity curves of the HyA at (**A**) 0.05 mg/mL, (**B**) 0.10 mg/mL, (**C**) 0.15 mg/mL and (**D**) 0.20 mg/mL (Filled markers: dilution; empty markers: uncorrected and CTAB-titrated HyA solution) (V_{HyA}= 19 mL, c_{CTAB}= 25 mM, high purified water medium).

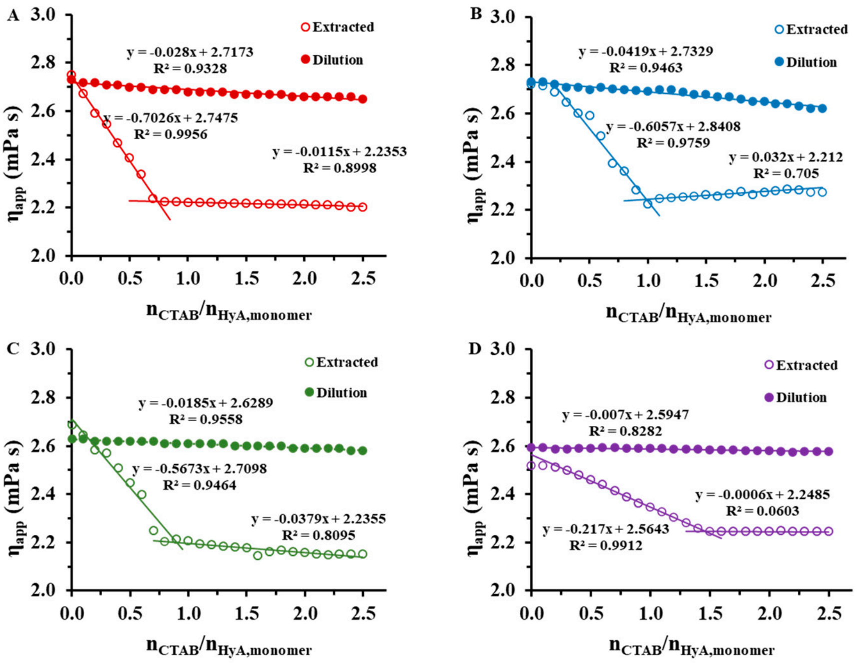

Figure S7. The apparent viscosity curves of the HyA at (**A**) pH=3.6, (**B**) pH=4.0, (**C**) pH=4.5 and (**D**) pH=5.5 acetate buffer (Filled markers: dilution; empty markers: uncorrected and CTAB-titrated HyA solution) (c_{HyA}= 0.10 mg/mL; V_{HyA}= 19 mL; c_{CTAB}= 25 mM).

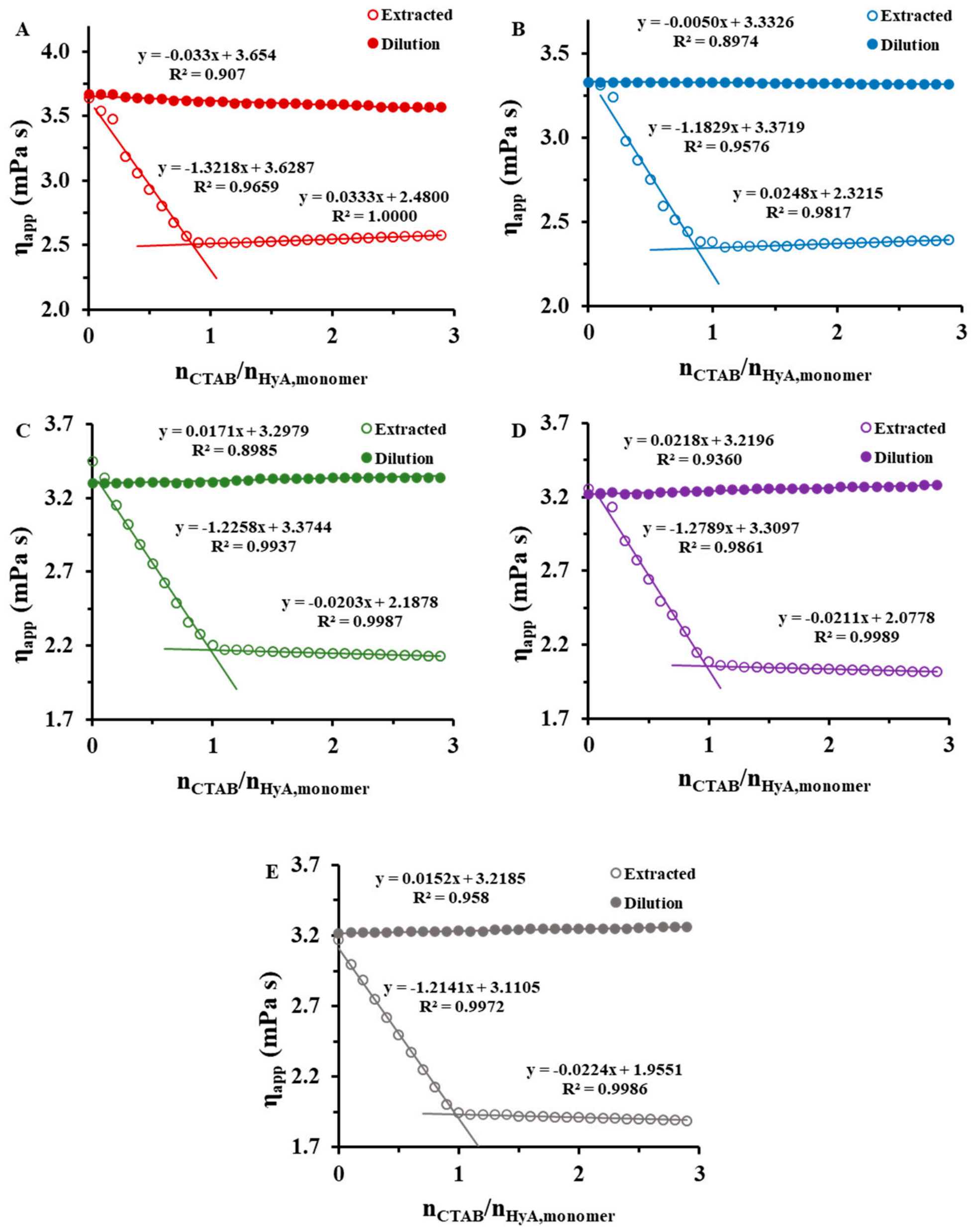

Figure S8. The apparent viscosity curves of the HyA at (**A**) 15°C, (**B**) 20°C, (**C**) 25°C, (**D**) 30°C and (**E**) 35°C (Filled markers: dilution; empty markers: uncorrected and CTAB-titrated HyA solution) (c_{HyA} = 0.10 mg/mL, V_{HyA}= 19 mL; c_{CTAB}= 25 mM, high purified water medium).

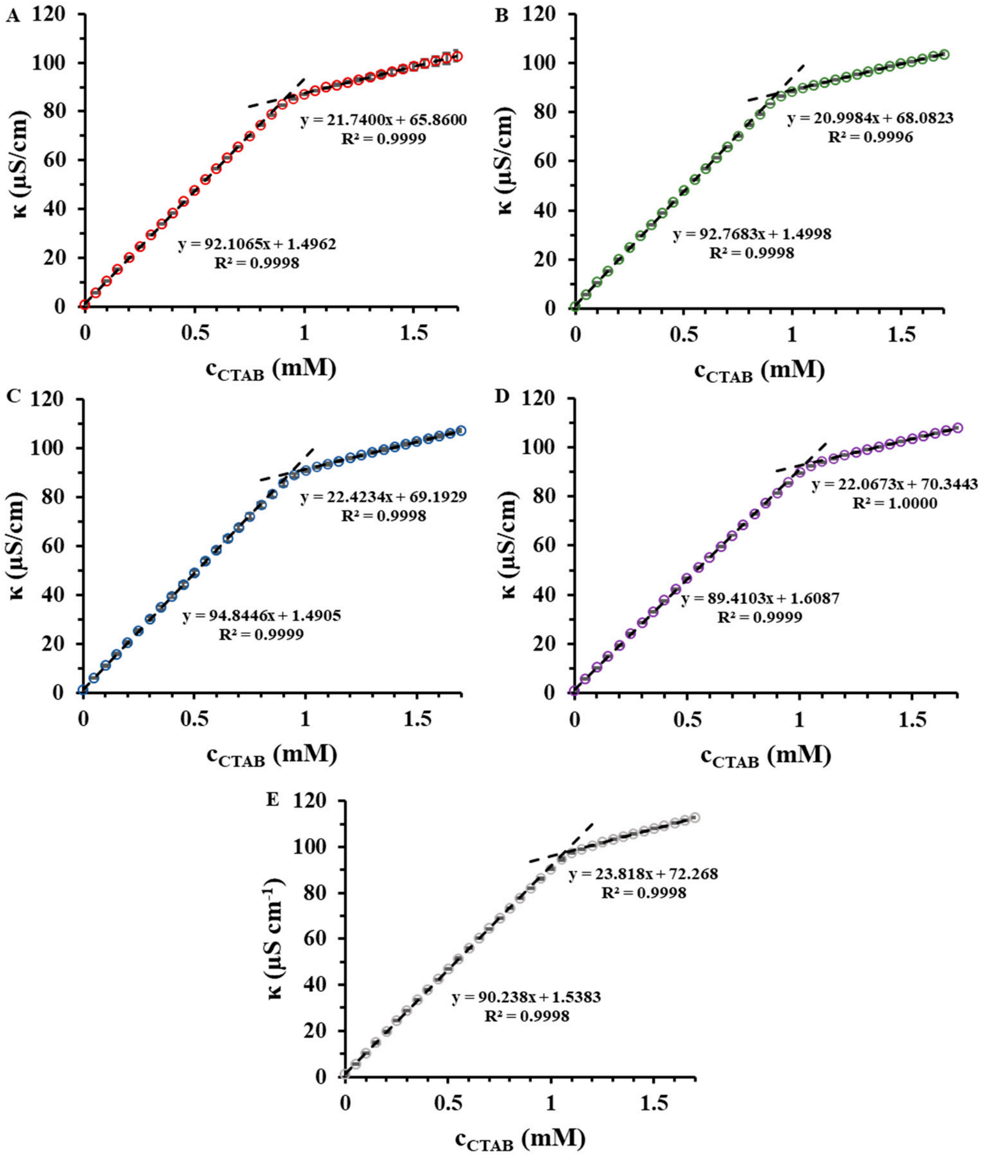

Figure S9. The conductometric titration of high purified water with CTAB (25 mM) at (**A**) 15°C, (**B**) 20°C, (**C**) 25°C, (**D**) 30°C and (**E**) 35°C temperature.
